# Semi-Quantitative Evaluation of BRCA1 Protein in Breast Tumors Using Anti-BRCA1 Antibodies: Clinical Implications

**DOI:** 10.3390/ijms27041729

**Published:** 2026-02-11

**Authors:** Sorana Caterina Anton, Alin Horatiu Nedelcu, Victor Ianole, Carmen Anton, Mihai Danciu, Ionela Daniela Morariu, Ancuța Lupu, Gabriel Dascalescu, Vasile Valeriu Lupu, Anton Knieling, Delia Nicolaiciuc, Alin Ciobica, Elena Andreea Chivasuta, Mihaela Tirnovanu, Iurie Dondiuc, Ciprian Ilea, Mihaela Grigore, Emil Anton

**Affiliations:** 1 Faculty of Medicine, “Grigore T. Popa” University of Medicine and Pharmacy, 700115 Iasi, Romaniaalin.nedelcu@umfiasi.ro (A.H.N.); mihai.danciu@umfiasi.ro (M.D.); ancuta.ignat1@umfiasi.ro (A.L.); vasile.lupu@umfiasi.ro (V.V.L.); emil.anton@umfiasi.ro (E.A.); 2 Department of Pathology, “Prof. Dr. Nicolae Oblu” Emergency Clinical Hospital, 700309 Iasi, Romania; 3 Department of Pathology, “Saint Spiridon” County Hospital, 700111 Iasi, Romania; 4 Faculty of Pharmacy, “Grigore T. Popa” University of Medicine and Pharmacy, 700115 Iasi, Romania; 5 Department of Biology, Faculty of Biology, Alexandru Ioan Cuza University of Iasi, B-Dul Carol I, No. 11, 700506 Iasi, Romania; gabidascalescu2001@gmail.com (G.D.);; 6 “Ioan Haulica” Institute, Apollonia University, 700511 Iasi, Romania; 7 CENEMED Platform for Interdisciplinary Research, “Grigore T. Popa” University of Medicine and Pharmacy, 700115 Iasi, Romania; 8 Biomedical Research Group, “Olga Necrasov” Center, Romanian Academy, Iasi Branch, Teodor Codrescu 2, 700481 Iasi, Romania; 9 Department of Obstetrics and Gynecology, “Nicolae Testemitanu” University of Medicine and Pharmacy, 2004 Chisinau, Moldova

**Keywords:** BRCA1, immunohistochemistry, triple-negative breast cancer, tumor microenvironment, biomarker

## Abstract

Aggressive breast cancer subtypes, such as triple-negative breast cancer (TNBC) and tumors in BRCA1 germline mutation carriers, present significant clinical challenges. The role of BRCA1 protein expression, assessed via immunohistochemistry (IHC), in defining the biology of these high-risk tumors remains to be fully elucidated. We performed a semi-quantitative IHC analysis of BRCA1 protein expression in a targeted cohort of 100 invasive breast carcinomas, enriched for TNBC (88%) and BRCA1 mutation carriers (34%). A validated monoclonal antibody and a composite scoring system (0–9) were employed, with an optimal cut-off defined by ROC analysis. Associations with clinicopathological parameters and p16 expression were evaluated. Low BRCA1 expression was strongly associated with an aggressive phenotype, including invasive ductal histology, hormone receptor negativity, and the TNBC subtype (all *p* < 0.001). Tumors with BRCA1 loss exhibited a more pro-tumorigenic microenvironment, characterized by higher rates of necrosis (*p* = 0.014) and denser mononuclear infiltrates (*p* = 0.019). A significant inverse correlation with p16 overexpression was identified (*p* = 0.030). Our findings demonstrate that BRCA1 protein loss delineates a distinct aggressive tumor biology within high-risk breast cancers. We emphasize that BRCA1 IHC is a complementary biomarker and cannot supplant germline genetic testing for clinical decision-making regarding targeted therapies.

## 1. Introduction

Breast cancer remains a major global health challenge, characterized by significant molecular and clinical heterogeneity [1]. Among its subtypes, triple-negative breast cancer (TNBC) and hereditary forms associated with germline BRCA1 mutations are particularly aggressive, posing substantial therapeutic challenges [1,2,3,4]. A deeper understanding of the molecular drivers in these high-risk cancers is crucial for improving patient stratification and outcomes.

The BRCA1 tumor suppressor protein is a central guardian of genomic integrity, playing multifaceted roles in DNA damage repair, cell cycle checkpoint control, and transcriptional regulation [5,6,7]. Its function is crucial for the homologous recombination repair of DNA double-strand breaks [5]. The loss of BRCA1 function, whether through inherited mutation or somatic alteration, leads to genomic instability and a high predisposition to carcinogenesis [8].

Carcinomas arising in BRCA1 germline mutation carriers typically exhibit a distinct phenotype, frequently presenting as high-grade estrogen receptor (ER)-, progesterone receptor (PR)-, and HER2-negative (TNBC) tumors with an aggressive clinical course [9,10]. Importantly, BRCA1 pathway dysfunction is not confined to hereditary cancers. In sporadic breast cancers, a “BRCA-like” phenotype can occur through mechanisms such as promoter hypermethylation or the loss of heterozygosity, leading to functional protein loss, particularly within the TNBC subtype [11,12].

The subcellular localization and prognostic significance of BRCA1 protein expression remain subjects of debate. Chen et al. reported that BRCA1 protein is found in the nuclei of normal epithelial cells but is aberrantly localized in the cytoplasm of malignant breast cells [13]. Furthermore, Jensen et al. reported that BRCA1 is localized in the cytoplasm and cell membrane [14], while Coene et al. proposed that it is located in a cytoplasmic tube-like invagination into the nucleus [15].

Immunohistochemistry (IHC) is a valuable tool for assessing BRCA1 protein status in tumor tissues. While earlier studies encountered challenges with antibody specificity and staining interpretation related to nuclear localization signals [16,17], the development of validated monoclonal antibodies has significantly improved the reliability of BRCA1 IHC in formalin-fixed, paraffin-embedded (FFPE) samples [18,19,20]. This allows for the robust identification of tumors with functional BRCA1 loss, which is a common feature in both hereditary and high-grade sporadic cancers [21,22,23].

Concurrently, the cell cycle regulator p16 has emerged as another key protein in aggressive breast cancer biology. p16 acts as a potent inhibitor of cell cycle progression and is frequently overexpressed in basal-like and TNBCs [24,25].

This study aimed to evaluate BRCA1 protein expression in tumor tissue using a validated monoclonal antibody. We employed a semi-quantitative scoring system assessing both the intensity and proportion of nuclear staining, which were evaluated at the level of the tumor parenchyma. These assessments are useful in clinical practice, serving as a surrogate marker for functional BRCA1 loss.

Therefore, this study was designed to (1) characterize BRCA1 protein expression using a validated antibody in a defined high-risk cohort and (2) correlate BRCA1 status with key clinicopathological parameters and tumor microenvironment features.

## 2. Results

### 2.1. Descriptive Analysis of the Study Cohort

The clinicopathological characteristics of the study cohort are summarized in Table 1. Consistent with the purposive sampling strategy, the cohort was enriched for aggressive features, with 88% triple-negative breast cancers and 84% invasive ductal carcinomas. The cohort encompassed a range of disease stages (stage I, 22%; stage II, 38%; stage III, 30%; and stage IV, 10%). BRCA1 protein expression, assessed using the composite score (0–9), showed a broad distribution across the cohort, enabling a meaningful correlative analysis.

### 2.2. Associations with Clinicopathological Features

**Figure 1:**
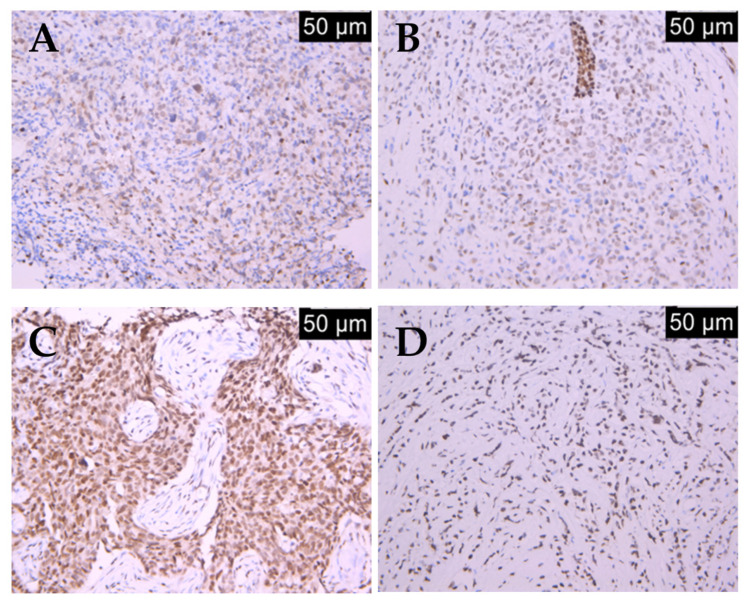
Low immunohistochemical expression levels of BRCA1 (IHC, anti-BRCA1 monoclonal antibody) in (**A**) invasive ductal carcinoma (IHC, ×20) and (**B**) invasive lobular carcinoma (IHC, ×20) versus high immunohistochemical expression levels of BRCA1 (IHC, anti-BRCA1 monoclonal antibody) in (**C**) invasive ductal carcinoma (IHC, ×20) and (**D**) invasive lobular carcinoma (IHC, ×20).

To provide a visual reference for semi-quantitative assessment, Figure 1 illustrates the distinct immunohistochemical patterns of BRCA1 protein expression observed across the cohort.

We first assessed the relationship between BRCA1 status and core clinicopathological parameters. No significant associations were found with patient age at diagnosis (*p* = 0.882). In contrast, strong correlations emerged with histology and molecular subtype. Low BRCA1 expression was overwhelmingly associated with invasive ductal carcinoma (93.4% of low-expressing cases), while high BRCA1 expression showed a significant association with invasive lobular carcinoma (41.7% of high-expressing cases; *p* < 0.001). Similarly, low BRCA1 expression was a hallmark of the TNBC subtype, present in 97.4% of low-expressing tumors, compared to 58.3% in the high-expression group, where luminal B tumors were more prevalent (37.5%, *p* < 0.001).

The analysis was extended to features of the tumor microenvironment. Tumors with low BRCA1 expression exhibited a significantly denser mononuclear inflammatory infiltrate (50.0% moderate-to-rich vs. 16.7%, *p* = 0.019) and a higher frequency of tumor necrosis (44.7% vs. 16.7%; *p* = 0.014). As anticipated from the molecular subtype distribution, low BRCA1 expression was strongly correlated with negative estrogen receptor (ER, *p* < 0.001) and progesterone receptor (PR, *p* < 0.001) status.

A key finding was the significant inverse correlation between BRCA1 and p16 expression, with high p16 levels detected in 73.7% of low-BRCA1 tumors vs. in 50.0% of high-BRCA1 tumors (*p* = 0.030, Table 2). No significant associations were observed for histological grade, lymphovascular invasion, perineural invasion, or HER2 status.

### 2.3. Relationship Between Germline BRCA1 Status and Protein Expression

We next examined the relationship between germline BRCA1 mutation status and tumoral protein expression in the 34 known mutation carriers. The following aspects were observed, with protein expression scores following a bimodal distribution. A substantial subset of tumors (41.2%) from mutation carriers retained high BRCA1 protein levels (score of 9, Table 3).

The specific germline mutation types analyzed (c.3067.C>T, c.5266dupC, and c.4035delA) were not correlated with the IHC score (*p* = 0.526, Table 4).

Within this genetic subgroup, clinicopathological features varied according to the BRCA1 protein score: tumors with the lowest scores (2–4) were exclusively TNBC ductal carcinomas, while those with the highest score (9) included older patients and a mix of histological and molecular subtypes (Table 5).

### 2.4. Univariate Analysis of BRCA1 Expression in Patients with Breast Carcinoma

Univariate analysis consolidated the strength of the observed associations (Table 6). The most robust correlations with low BRCA1 expression were confirmed for invasive ductal histology (Cramer’s V = 0.551), the triple-negative subtype (V = 0.527), and hormone receptor negativity (ER: V = 0.515; PR: V = 0.438; all *p* < 0.001). Significant, albeit weaker, correlations were maintained for mononuclear infiltrate density (V = 0.303, *p* = 0. 019), tumor necrosis (V = 0.247, *p* = 0.014), and p16 overexpression (V = 0.217, *p* = 0.030).

**Figure 2:**
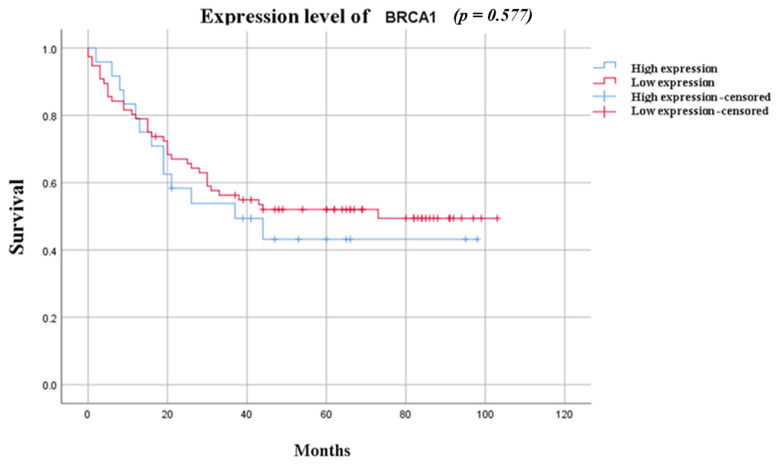
Kaplan–Meier survival curve in relation to BRCA1 expression level.

Finally, we evaluated clinical outcomes. Kaplan–Meier analysis revealed no significant difference in overall survival between patients with low vs. high BRCA1 protein expression in this heterogeneous cohort (log-rank test, *p* = 0.577, Figure 2).

Although we identified a significant biological inverse correlation between BRCA1 loss and p16 overexpression (*p* = 0.030), this relationship did not translate into a distinct survival benefit. Specifically, the Kaplan–Meier survival analysis stratified by p16 status (high vs. low) revealed no statistically significant difference in overall survival within this cohort (Log-rank test, *p* = 0.966). These findings suggest that, while p16 upregulation is a hallmark feature of the BRCA1-deficient phenotype, it does not serve as an independent prognostic stratification marker in this specific study population.

## 3. Discussion

This study delineates the clinicopathological characteristics associated with BRCA1 protein expression in a high-risk breast cancer cohort. Our results confirm and extend previous findings by demonstrating that the loss of BRCA1 expression, evaluated through a validated immunohistochemical approach, defines an aggressive tumor phenotype characterized by triple-negative status, hormone receptor negativity, and a pro-inflammatory tumor microenvironment. We further identified a significant inverse association between BRCA1 expression and p16 overexpression, highlighting the complex interaction between DNA damage response mechanisms and cell cycle regulation. Importantly, our findings emphasize that BRCA1 immunohistochemistry serves as a complementary research tool and cannot replace genetic testing in clinical decision-making.

Low BRCA1 expression was observed in 71% of invasive ductal carcinomas, a predominance that was statistically significant (*p* < 0.001). This observation aligns with previous reports suggesting that BRCA1 downregulation contributes to genomic instability and tumor progression in ductal breast cancer subtypes [26,27]. BRCA1 plays a critical role in homologous recombination-mediated repair of DNA double-strand breaks, and its deficiency allows the accumulation of genetic damage, thereby promoting malignant transformation [28,29]. Clinically, BRCA1-deficient invasive ductal carcinomas are frequently associated with higher mitotic rates and poorer differentiation, consistent with the more aggressive histological features observed in our BRCA1-low group [30,31].

The inverse association between BRCA1 expression and invasive lobular carcinoma should be interpreted with caution due to the limited number of lobular cases (*n* = 11) in our cohort. Validation in larger, subtype-focused cohorts is required before definitive conclusions can be drawn.

A strong inverse correlation was identified between BRCA1 expression and hormone receptor status: 90% of estrogen receptor-negative and 92% of progesterone receptor-negative tumors exhibited low BRCA1 expression (*p* < 0.001 for both). These findings support experimental evidence showing that BRCA1 regulates estrogen receptor transcriptional activity, with its loss favoring hormone-independent tumor growth [13]. In addition, Foulkes et al. (2010) demonstrated that triple-negative breast cancers frequently exhibit BRCA1 dysfunction, explaining the close association between BRCA1 loss and hormone receptor negativity [31]. While this relationship underscores the aggressive nature of BRCA1-deficient tumors, BRCA1 expression alone should not be interpreted as a surrogate marker of endocrine therapy resistance, which must be defined by clinical treatment response.

Analysis by molecular subtype revealed that low BRCA1 expression was most prevalent in triple-negative breast cancer (84% of cases), whereas luminal B tumors largely retained BRCA1 expression (90%). This distribution is consistent with prior proteomic and genomic studies demonstrating subtype-specific regulation and the loss of BRCA1 [32,33]. Although BRCA1 immunohistochemistry provides valuable biological insight, it cannot replace genetic testing when guiding therapies targeting DNA repair deficiencies such as platinum-based chemotherapy or PARP inhibitors. Nevertheless, BRCA1 IHC may complement next-generation sequencing by offering functional information on protein expression, potentially aiding in the identification of BRCA1-like phenotypes in sporadic tumors.

Tumor necrosis was significantly more frequent in BRCA1-low tumors (44.7% vs. 16.7%; *p* = 0.014), and dense mononuclear inflammatory infiltrates were also more common (*p* = 0.019). Tumor necrosis is generally associated with high proliferative activity and hypoxia, features linked to poor prognosis. BRCA1 loss may exacerbate proliferative stress, thereby promoting necrosis, as previously suggested by Wilson et al. [23]. Additionally, BRCA1-deficient tumors may foster a pro-inflammatory microenvironment through cytokine signaling, further contributing to genomic instability and tumor progression.

From a mechanistic standpoint, the pronounced inflammatory landscape associated with BRCA1-low tumors may be driven by the activation of the cGAS-STING cytosolic DNA-sensing pathway [34]. When BRCA1 function is compromised, genomic instability leads to the accumulation of cytosolic DNA species. These aberrant nucleic acids are detected by cGAS (cyclic GMP–AMP synthase), which subsequently triggers a STING-dependent type I interferon response [35]. This signaling cascade effectively recruits mononuclear immune cells to the tumor microenvironment, explaining the dense inflammatory infiltrate that we observed in samples with lost BRCA1 expression.

A notable finding was the association between low BRCA1 expression and high p16 immunostaining (*p* = 0.030), observed in approximately half of BRCA1-deficient tumors. p16 functions as a cyclin-dependent kinase inhibitor involved in G1/S cell cycle arrest in response to oncogenic or DNA damage stress [25]. In the context of BRCA1 loss, p16 overexpression may represent a compensatory attempt to restrain cell cycle progression; however, this checkpoint is likely insufficient to counteract the extensive genomic instability characteristic of BRCA1-deficient tumors. Despite this association, p16 expression cannot substitute for genomic testing when selecting patients for PARP inhibitor therapy.

Kaplan–Meier analysis revealed no significant difference in overall survival between the BRCA1-low and BRCA1-high groups (*p* = 0.577). This finding is consistent with that of prior studies indicating that BRCA1 status is predictive of response to DNA-damaging therapies rather than an independent prognostic factor in heterogeneous patient populations [31]. Treatment variability, adjuvant therapies, and additional genetic alterations may confound the relationship between BRCA1 expression and survival outcomes [36].

Methodologically, BRCA1 expression was assessed using a rabbit monoclonal antibody (Abcam ab213929) with optimized antigen retrieval and dual-pathologist scoring. ROC analysis demonstrated strong discriminative performance (AUC = 0.857). Although concerns regarding BRCA1 antibody specificity and subcellular localization persist [16,17], internal validation using control tissues with known BRCA1 status supported the reliability of our staining protocol. Nonetheless, continued standardization of pre-analytical and analytical parameters remains essential for cross-study comparability.

While BRCA1 IHC cannot replace molecular testing for identifying pathogenic variants, our data support its role as a complementary biomarker that provides functional insight into tumor biology, particularly in triple-negative breast cancer. Combined BRCA1 and p16 validation may further refine biological risk stratification by identifying tumors under heightened replicative stress. Although derived from Romanian centers, the clinicopathological associations observed in this study are consistent with international data, suggesting that the interplay between BRCA1 loss and aggressive tumor behavior is broadly generalizable [37].

Looking beyond protein expression, the aggressive clinical behavior of TNBC is frequently exacerbated by p53 pathway dysfunction and the dysregulation of non-coding RNAs, both of which contribute to chemoresistance. Consequently, the therapeutic landscape is shifting towards precision medicine approaches that can overcome these barriers. Emerging research highlights the potential of nanomedicine, specifically engineered exosomes, as novel gene therapy vectors. These nanocarriers are being investigated for their ability to deliver tumor-suppressor genes or therapeutically target metastatic niches, offering a promising complementary strategy to standard regimens for high-risk, BRCA1-deficient patients [38,39].

A key characteristic of our study design was the intentional enrichment of the cohort with triple-negative breast cancer and BRCA1 mutation carriers to specifically investigate these high-risk phenotypes. Due to this purposive sampling strategy, the prevalence of TNBC (88%) and BRCA1 mutations (34%) in our dataset is significantly higher than that observed in the general breast cancer population. While this approach strengthened our ability to analyze aggressive subtypes, it inherently implies that our findings are most applicable to high-risk cohorts rather than the unselected general population. Furthermore, the study size of 100 cases, while valuable for this targeted analysis, warrants future validation in larger, external datasets (such as TCGA) to enhance generalizability. Additionally, given that our cohort is predominantly of Eastern European descent, the results may not fully reflect the biological variations present in populations of African descent, where TNBC prevalence is naturally higher.

Regarding molecular characteristics, we lacked data on somatic BRCA1 mutations and promoter methylation status. Moreover, as the study period predated the widespread reimbursement of PARP inhibitors in our region, we could not evaluate the predictive value of BRCA1 protein levels for PARP inhibitor response, representing a critical avenue for future prospective research.

## 4. Materials and Methods

### 4.1. Study Design and Patient Cohort

This retrospective cross-sectional study analyzed BRCA1 protein expression in a consecutively recruited cohort of 100 patients diagnosed with invasive breast carcinoma between January 2015 and December 2024. Patients were identified from two tertiary care centers in Romania: the Regional Institute of Oncology, Iasi, and the “Cuza Voda” Clinical Hospital of Obstetrics and Gynecology, Iasi. Our study included cases with a triple-negative breast cancer (TNBC) phenotype and/or a known germline BRCA1 mutation status. Consequently, the cohort demonstrates a high prevalence of TNBC (88%) and germline BRCA1 mutation carriers (34%), which is not representative of an unselected breast cancer population but is optimally designed for investigating correlations within these high-risk subgroups. Patients with both early-stage (I–II) and advanced-stage (III–IV) disease were included. The study required complete clinicopathological documentation and sufficient archival tumor tissues; cases lacking these were excluded.

### 4.2. Immunohistochemistry (IHC) Protocols

Immunohistochemical analysis of BRCA1 was performed on 4 µm formalin-fixed, paraffin-embedded (FFPE) tissue sections. After heat-induced epitope retrieval in pH 9 buffer (96 °C, 25 min), sections were incubated with a validated monoclonal rabbit anti-BRCA1 antibody (dilution 1:400, ab213929, Abcam, Cambridge, UK). Detection was achieved using the UltraVision LP Detection System (Thermo Fisher Scientific, Fremont, CA, USA) with 3,3′-diaminobenzidine (DAB) as the chromogen. Rigorous internal validation was performed in each staining batch, incorporating positive controls (normal breast epithelium with known nuclear staining) and negative controls (a formalin-fixed, paraffin-embedded block of known BRCA1-defficient cell line and primary antibody omission). The resulting staining was predominantly nuclear. Immunostaining for estrogen receptor (ER), progesterone receptor (PR), HER2, and p16 was performed as part of routine diagnostic procedures according to established institutional protocols. Microscope evaluation and image acquisition were performed using a Leica DM3000 LED light microscope (Leica Biosystems, Nußloch, Germany). 

### 4.3. Scoring System and Interpretation

To ensure the objectivity and reproducibility of the results, the immunohistochemical evaluation was conducted in a double-blind manner by two independent, experienced pathologists who were unaware of the patient’s clinical, pathological, and genetic data. The inter-observer agreement was statistically substantial, yielding a Cohen’s kappa coefficient of 0.82.

BRCA1 immunohistochemical nuclear staining of tumor cells was considered positive. The percentage of positive tumor cells and the intensity of the immunoreaction were assessed using a four-tiered scoring system as follows: 0 = 0% positive tumor cells, 1 = 1–20% positive tumor cells, 2 = 21–80% positive tumor cells, and 3 = 81–100% positive tumor cells; 0 = negative reaction, 1 = weak intensity, 2 = moderate intensity, and 3 = strong intensity. The proportion and intensity scores were multiplied to determine the total immunoreactivity score (IRS). Consequently, the IRS varied between 0 and 9. The immunoreaction was assessed in the tumor core and invasive front.

To evaluate BRCA1 immunohistochemical expression as low/high, a cut-off point for the IRS was established using receiver operating characteristic (ROC) curve analysis by maximizing Youden’s index. Therefore, a cut-off value of 6 was found (IRS ≤ 6—low immunohistochemical expression/IRS > 6—high immunohistochemical expression).

p16 immunohistochemical nuclear and cytoplasmatic staining of tumor cells was considered positive. The intensity of the immunoreaction was assessed in the nuclei and cytoplasm using a four-tiered scoring system as follows: 0 = negative reaction, 1 = weak intensity, 2 = moderate intensity, and 3 = strong intensity. The intensity nuclear score and cytoplasmatic nuclear score were summed to determine the total immunoreactivity score (IRS). Consequently, the IRS varied between 0 and 6. The immunoreaction was assessed in the tumor core and invasive front.

To evaluate p16 immunohistochemical expression as low/high, a cut-off point for the IRS was established using receiver operating characteristic (ROC) curve analysis by maximizing Youden’s index. Therefore, a cut-off value of 2 was found (IRS ≤ 2—low immunohistochemical expression/IRS > 2—high immunohistochemical expression).

Hormone receptor status (ER and PR) was stratified into three categories: negative (<1% stained cells), positive (≥1%), and intensely positive (strong nuclear staining in >50% of cells).

### 4.4. Clinicopathological Parameters and Statistical Analysis

Clinicopathological data extracted from medical records included age; histological type and grade; ER, PR, and HER2 status; molecular subtype (luminal A/B, HER2-enriched, or TNBC); tumor necrosis; mononuclear inflammatory infiltrate density; and p16 status. Statistical analyses were performed using SPSS Statistics version 31.0.1.0 (IBM Corp., Armonk, NY, USA). Associations between dichotomized BRCA1 expression and categorical variables were assessed using Chi-square test or Fisher’s exact test, as appropriate. The correlation strength for significant associations is reported using Cramer’s V. Overall survival was analyzed with Kaplan–Meier curves and compared using the log-rank test. A two-sided *p*-value < 0.05 was considered statistically significant.

### 4.5. Ethical Considerations

This study was conducted following the ethical principles of the Declaration of Helsinki. Approval was granted by the Ethics Committees of the Regional Institute of Oncology Iasi (925/20 October 2021) and “Grigore T. Popa” University of Medicine and Pharmacy Iasi (379/17 January 2024). The retrospective nature of the study, utilizing anonymized archival data and tissue, permitted a waiver of individual informed consent. Data handling complied with the European Union General Data Protection Regulation (GDPR).

## 5. Conclusions

Our findings demonstrate that the loss of BRCA1 protein expression, as assessed via IHC, is indicative of an aggressive tumor phenotype in high-risk breast cancer, characterized by triple-negative status, hormone receptor negativity, and a pro-inflammatory necrotic tumor microenvironment. A novel aspect of this study is the significant inverse association between BRCA1 loss and p16 overexpression, indicating concomitant disruption of DNA repair mechanisms and cell cycle regulation.

Importantly, BRCA1 IHC should be regarded as a complementary research tool and not as a substitute for germline genetic testing in clinical decision-making, particularly for determining eligibility for PARP inhibitor therapy. Future studies should validate the combined BRCA1/p16 immunophenotype as a biological stratification marker in aggressive breast cancer subtypes, especially triple-negative disease, and assess its potential value in predicting response to DNA-damaging treatments in prospective, unselected cohorts.

## Figures and Tables

**Table 1:** Baseline clinicopathological characteristics of the study cohort (*n* = 100).

Characteristic	Category	*n* (%)
Age	<50 Years	25 (25%)
	50–70	45 (45%)
	>70 Years	30 (30%)
Histological Type	Invasive Ductal Carcinoma	84 (84%)
	Invasive Lobular Carcinoma	11 (11%)
	Other	5 (5%)
Molecular Subtype	Triple-Negative	88 (88%)
	Luminal B	10 (10%)
	HER2-Positive	2 (2%)
Receptor Status	ER-Negative	90 (90%)
	PR-Negative	92 (92%)
	HER2-Negative	95 (95%)
Tumor Stage	I	22 (22%)
	II	38 (38%)
	III	30 (30%)
	IV	10 (10%)

**Table 2:** BRCA1 expression vs. p16 protein expression.

	BRCA1 Low (*n* = 76)	BRCA1 High (*n* = 24)	Total (*n* = 100)	Statistics
p16 Low	20 (26.3%)	12 (50.0%)	32	*p*-value: 0.030
p16 High	56 (73.7%)	12 (50.0%)	68	Cramer’s V: 0.217

**Table 3:** Distribution of germline BRCA-mutated patients according to BRCA1 score values.

SCORE	*n*	%
2	2	5.9%
3	6	17.6%
4	2	5.9%
6	10	29.4%
9	14	41.2%
Total	34	100.0%

**Table 4:** Score values depending on BRCA1 mutation type.

BRCA1 Score	c.3067.C>T (*n* = 24)	c.5266dupC (*n* = 6)	c.4035delA (*n* = 4)	Statistical Test
2	2 (8.3%)	0 (0.0%)	0 (0.0%)	Chi-square X^2^ = 7.097 *p*-value = 0.526
3	5 (20.8%)	0 (0.0%)	1 (25.0%)	
4	2 (8.3%)	0 (0.0%)	0 (0.0%)	
6	7 (29.2%)	1 (16.7%)	2 (50.0%)	
9	8 (33.3%)	5 (83.3%)	1 (25.0%)	
Total	24 (100%)	6 (100.0%)	4 (100.0%)	

**Table 5:** Comparative immunolabeling analysis according to score values.

Characteristic	Score 2 (*n* = 2)	Score 3 (*n* = 6)	Score 4 (*n* = 2)	Score 6 (*n* = 10)	Score 9 (*n* = 14)	*p*-Value
Age group						
<50 years	1 (50.0%)	2 (33.3%)	1 (50.0%)	2 (20.0%)	2 (14.3%)	0.060
50–60 years	1 (50.0%)	1 (16.7%)	0 (0.0%)	5 (50.0%)	0 (0.0%)	
>60 years	0 (0.0%)	3 (50.0%)	1 (50.0%)	3 (30.0%)	12 (85.7%)	
Histology						
Ductal	2 (100.0%)	5 (83.3%)	2 (100.0%)	9 (90.0%)	7 (50.0%)	0.497
Lobular	0 (0.0%)	1 (16.7%)	0 (0.0%)	1 (10.0%)	6 (42.9%)	
Mixed	0 (0.0%)	0 (0.0%)	0 (0.0%)	0 (0.0%)	1 (7.1%)	
ER Status						
Negative	2 (100.0%)	6 (100.0%)	2 (100.0%)	9 (90.0%)	9 (64.3%)	0.628
Positive	0 (0.0%)	0 (0.0%)	0 (0.0%)	1 (10.0%)	3 (21.4%)	
High Positive	0 (0.0%)	0 (0.0%)	0 (0.0%)	0 (0.0%)	2 (14.3%)	
PR Status						
Negative	2 (100.0%)	6 (100.0%)	2 (100.0%)	9 (90.0%)	10 (71.4%)	0.776
Positive	0 (0.0%)	0 (0.0%)	0 (0.0%)	1 (10.0%)	2 (14.3%)	
High Positive	0 (0.0%)	0 (0.0%)	0 (0.0%)	0 (0.0%)	2 (14.3%)	
HER 2 Status						
Negative	2 (100.0%)	6 (100.0%)	2 (100.0%)	9 (90.0%)	13 (92.9%)	0.914
Positive	0 (0.0%)	0 (0.0%)	0 (0.0%)	1 (10.0%)	1 (7.1%)	

**Table 6:** Univariate analysis of BRCA1 expression and clinicopathological parameters in patients with breast carcinoma.

Clinicopathological Parameters		*n*	BRCA1 Expression		*p*-Value	Cramer’s V
			Low	High		
Age	31–40	5	5 (5%)	0 (0%)	0.822	0.167
	41–50	10	8 (8%)	2 (2%)		
	51–60	22	17 (17%)	5 (5%)		
	61–70	20	16 (16%)	4 (4%)		
	71–80	30	21 (21%)	9 (9%)		
	81–90	13	9 (19%)	4 (4%)		
Histological type	Invasive ductal carcinoma	84	71 (71%)	13 (13%)	0.000	0.551
	Invasive lobular carcinoma	11	1 (1%)	10 (10%)		
	Other	5	4 (4%)	1 (1%)		
Histological grade	G1	0	-	-	0.424	0.384
	G2	2	1 (1%)	1 (1%)		
	G3	98	75 (75%)	23 (23%)		
Lymphovascular invasion	Absent	83	63 (63%)	20 (20%)	1.000	0.005
	Present	17	13 (13%)	4 (4%)		
Perineural invasion	Absent	95	72 (72%)	23 (23%)	1.000	0.021
	Present	5	4 (4%)	1 (1%)		
Mononuclear inflammatory infiltrate	Absent	29	19 (19%)	10 (10%)	0.019	0.303
	Mild	29	19 (19%)	10 (10%)		
	Moderate	30	26 (26%)	4 (4%)		
	Rich	12	12 (12%)	0 (0%)		
Tumor necrosis	Absent	62	42 (42%)	20 (20%)	0.014	0.247
	Present	38	34 (34%)	4 (4%)		
Estrogen receptor status	Absent	90	75 (75%)	15 (15%)	0.000	0.515
	Present	10	1 (1%)	9 (9%)		
Progesterone receptor status	Absent	92	75 (75%)	17 (17%)	0.000	0.438
	Present	8	1 (1%)	7 (7%)		
HER2 status	Absent	95	74 (74%)	21 (21%)	0.088	0.193
	Present	5	2 (2%)	3 (3%)		
Molecular subtype	Luminal B	10	1 (1%)	9 (9%)	0.000	0.527
	HER2-positive	2	1 (1%)	1 (1%)		
	Triple-negative	88	74 (74%)	14 (14%)		
p16 expression	Low	32	20 (20%)	12 (12%)	0.030	0.217
	High	68	56 (56%)	12 (12%)		

*n* = total number of cases, *p*-value < 0.05.

## Data Availability

The original contributions presented in this study are included in the article. Further inquiries can be directed to the corresponding author.
